# Prolonged Grief Disorder Among Refugees in Psychological Treatment—Association With Self-Efficacy and Emotion Regulation

**DOI:** 10.3389/fpsyt.2020.00526

**Published:** 2020-06-05

**Authors:** Oriane Lacour, Naser Morina, Julia Spaaij, Angela Nickerson, Ulrich Schnyder, Roland von Känel, Richard A. Bryant, Matthis Schick

**Affiliations:** ^1^Department of Consultation-Liaison-Psychiatry and Psychosomatic Medicine, University Hospital, Zurich, University of Zurich, Zurich, Switzerland; ^2^School of Psychology, University of New South Wales, Sydney, NSW, Australia; ^3^Faculty of Medicine, University of Zurich, Zurich, Switzerland

**Keywords:** refugees, refugee mental health, prolonged grief disorder, self-efficacy, emotion regulation, trauma, post-migration living difficulties

## Abstract

**Background:**

While Prolonged Grief Disorder (PGD) among refugees has recently started to attract scientific attention, knowledge regarding associated psychological factors remains limited. Given the multifactorial context of persecution, trauma, displacement, and exile-related difficulties, obtaining a better understanding of PGD in refugees is crucial because PGD may affect psychological well-being, level of functioning, and social integration.

**Methods:**

In a sample of refugees receiving psychological treatment in Switzerland (*N* = 88), we examined the relationship between severity of PGD and potentially associated factors such as emotion regulation, perceived self-efficacy, as well as potentially traumatic events and post-migration living difficulties.

**Results:**

In a regression analysis, difficulties in emotion regulation and lower perceived self-efficacy were associated with greater severity of PGD, while post-migration living difficulties and potentially traumatic events did not emerge as significant factors.

**Conclusion:**

These findings suggest that emotion regulation and perceived self-efficacy are associated with PGD in refugees in psychological treatment and are thus potential targets for treatment interventions.

## Introduction

There are currently 70.8 million displaced people worldwide as a consequence of violence, persecution, conflict, or human rights violation (1). Forcibly displaced people frequently experience interpersonal loss and bereavement along with potentially traumatic events (PTE), including interpersonal violence, torture, life-threatening injuries and witnessing the death of loved ones (2). Accordingly, mental health issues such as posttraumatic stress disorder (PTSD), depression or anxiety disorders are prevalent (3).

While trauma and trauma-related disorders in forcibly displaced populations have received consistent scientific attention, research on loss and grief in this group is scarce (3–5). Prolonged Grief Disorder (PGD) has recently been recognized as a clinically relevant mental health issue which will be included as a new diagnosis in the upcoming *International Classification of Diseases, 11^th^ Revision* (ICD-11) (6). PGD, otherwise known as complicated grief (CG), refers to a distinct set of symptoms following the loss of a loved one. Those affected generally suffer from a persistent and pervasive longing for or preoccupation with the deceased, accompanied by emotional distress and significant functional impairment persisting more than six months after the loss. In the *Fifth Edition of the Diagnostic and Statistical Manual of Mental Disorder* (DSM-5), similar symptoms were classified as Persistent Complex Bereavement Disorder (PCBD). The latter differs from the former primarily with regard to the duration of symptoms (at least twelve months) (7). Due to the heterogeneous criteria used to assess CG, PGD, and PCBD in the past three decades, reported prevalence rates differ significantly. Yet, it is estimated that one out of ten bereaved individuals is at risk for disturbed grief following a nonviolent death (8) and that this risk is even higher after an unnatural (sudden, violent) death, affecting five out of ten bereaved individuals (9). Consequently, those individuals may experience substantial psychological distress, impairments in work and social functioning (7, 10, 11), and reduced quality of life (12, 13).

In refugees and post-conflicts samples, the prevalence of PGD has been estimated to be approx. 30% with important variation depending on cultural background (14, 15). Nevertheless, as only few studies with heterogeneous methodology have investigated PGD in refugees, the reported rates in this group also substantially differ from one another. Whereas earlier studies investigating PGD in refugees have focused on the associated comorbidity, traumatic experiences, and sociodemographic factors, as well as on cross-cultural adequacy of the concept, understanding of PGD and its associated factors is incomplete. As in the general population (7, 16, 17), both traumatic loss and violent loss have emerged as important predictors of PGD in refugees (18, 19). Similarly, strong associations with murder or disappearance of a family member have been described, as well as associations with other PTE such as torture or combat exposure (20). Nevertheless, the existence of a dose–response relationship between PTE exposure and PGD is currently inconclusive. Whereas exposure to a greater number of PTE might increase the likelihood of PGD diagnosis (20), some studies have found that trauma exposure is either not a significant predictor of PGD (21, 22) or not associated with PGD symptom severity (23). Furthermore, there seems to be no association between cumulated loss and PGD diagnosis (21, 22) or symptom severity (19), although multiple loss has been associated with anxiety, depression, disability, and worse quality of life in bereaved refugees (24).

While trauma seems to be related to PGD, it is likely that other contributing factors exist. For instance, nontraumatic stressors such as difficulties arising after arrival in a safe host country—referred to as Post-Migration Living Difficulties (PMLD)—might contribute to the likelihood of PGD diagnosis (20, 25, 26) and PGD symptom level (23). In addition, in nonrefugee samples, regulatory emotional–cognitive processes have been described as conclusive underlying mechanisms of adaptive coping with bereavement (27, 28). For example, negative cognitions (*i.e.* negative beliefs about self, world, life, future, others) and distressing interpretation of grief reactions (*e.g.* “If I let go of my emotions, I will go crazy”) have been found to be strongly related to higher concurrent and prospective levels of complicated grief symptoms, and to influence symptom severity over and above sociodemographic and loss-related variables (29, 30). Furthermore, these cognitive processes seem to be important correlates to emotional problems and maladaptive reactions to loss (30). On the other hand, factors such as optimism, active coping, and help seeking, as well as a supportive social environment, appear to be associated with less severe symptoms of PGD (31). Given the particular life circumstances refugees are confronted with, including displacement, exposure to multiple PTE and PMLD, it is unclear whether the findings from the general population can be generalized to refugees.

In view of the high prevalence of PGD in refugees and its potentially detrimental effects on psychological wellbeing, level of functioning, and social integration in the host country, it is crucial to acquire a better understanding of PGD to also provide appropriate treatment. This requires not only the assessment of risk factors, but also an exploration of protective factors. Surprisingly, the latter have not been emphasized in previous studies, even though they could be highly relevant for treatment interventions. Whereas risk factors of PGD such as loss-related characteristics cannot be modified through therapy, individual-related protective and adaptive factors could constitute promising treatment targets. This follows the idea that refugees and asylum seekers can benefit from resilience-focused interventions, along with trauma-focused interventions (32). The aim of this cross-sectional study was therefore to explore the relationship between the severity of PGD in a clinical sample of refugees and associated emotional and cognitive factors, specifically perceived self-efficacy and emotion regulation, with the hypothesis that they contribute to important mechanisms of adaptation in grief.

Perceived self-efficacy refers to positive beliefs about one's abilities to influence events that affect one's life. These beliefs may alter personal goals, motivation, everyday functioning, resilience to adversity, and vulnerability to distress and depression (33). The role of self-efficacy in adaptation after both a violent (34) and nonviolent loss of a loved one (35, 36) has been established in nonrefugee samples. Whereas self-efficacy has been considered a relevant variable among refugees due to its positive association with mental health and well-being (37, 38), its relationship to PGD remains unknown. Emotion regulation can be defined as one's ability to monitor, assess, and modulate experienced emotions in order to facilitate adaptive functioning (39). In this study, we refer to emotion regulation as conceptualized by Gratz and Roemer and involving the following features: awareness, understanding and acceptance of emotions, as well as impulse control and ability to modulate emotional responses and behaviors according to desired goals and situational demands (39). As grief is characterized by a wide range of strong, often negative emotions, difficulties in emotion regulation or active avoidance of grief-related emotions might lead to maladaptive bereavement responses (11, 40). A small number of studies among refugees suggested that emotion regulation mediates the relationship between trauma and psychological symptoms (41) and that difficulties in emotion regulation are associated with more severe symptoms of PTSD, depression, and anxiety (42). Conversely, the relationship between emotion regulation and PGD has not yet been explored in refugees.

Based on this literature, we hypothesized that perceived self-efficacy and emotion regulation would be associated with the severity of PGD in refugees in the host country Switzerland. We included reported PMLD and PTE in our exploration as relevant variables among refugees and potential further associated factors.

## Methods

### Participants

Participants were 88 refugees and asylum seekers receiving psychological treatment in two outpatient clinics for victims of torture and war in Bern and Zürich, Switzerland. At the time of inclusion, participants were at different stages of treatment. They were seeking treatment for varied psychopathology. Depending on their symptom profiles and subjective needs, treatment delivered included trauma-specific therapy, but also nonspecific psychotherapy, medication, and social counseling. Participants aged 18 or older and speaking either German, English, Turkish, Farsi, Tamil, or Arabic were included. Exclusion criteria were inability to use the tablet-based software used to collect data or inability to complete self-reported questionnaires, psychotic symptoms, severe dissociative symptoms, and acute suicidality. Based on the inclusion criteria, 106 patients were invited to participate in the study and were informed through a therapist and a study team member. In total, 88 patients (87%) agreed to participate. Reasons for rejecting were mainly: no interest and no time.

### Measures

If available, validated instruments in the specific language were used. All other instruments were translated and back-translated by accredited translators in accordance with gold-standard translation practices (43). Discrepancies were rectified jointly by the research team and independent bilingual individuals who were experienced in working with health-related questionnaires.

PGD was assessed using the Prolonged Grief Disorder-13 (PG-13) scale based on the PGD-2009 criteria proposed by Prigerson and colleagues (10). This scale contains 13 items: two yes–no items on duration and impairment and eleven items assessing cognitive, behavioral, and emotional symptoms, rated on a 5-point scale (either a frequency scale ranging from “not at all” to “several times a day”, or an intensity scale ranging from “not at all” to “overwhelmingly”). To assess the likelihood of PGD diagnosis five criteria must be considered: (A) event: the respondent has experienced the loss of a loved one; (B) separation distress: grief-related yearning is experienced at least daily; (C) duration: symptoms of separation distress remain at least 6 months after the loss; (D) cognitive, emotional, and behavioral symptoms: at least five symptoms based on items 4–11 are experienced at least “once a day” or “quite often”; (E) impairment: the respondent must have significant impairment in social, occupational, or other important areas of functioning. PG-13 can be scored as a continuous variable by summing the symptom items after excluding the two yes/no items about duration and functional impairment (44). This total score (range 11 to 55) was used as a severity score of PGD in the present study. To our knowledge, there are no universally recommended cut-off scores for clinical purposes. Internal consistency for this scale was *α* = 0.88 in our sample.

Overall trauma exposure was assessed combining the Harvard Trauma Questionnaire (HTQ) (45) and the Posttraumatic Diagnostic Scale (46) resulting in a total of 23 items. Items include for example: “serious accident, fire or explosion”, “being close to death”, “imprisonment”. Potentially traumatic events experienced by each participant were summed up to a total count. In addition, loss-related characteristics were derived from the items “murder of a family member or friend” and “unnatural death of a family member or friend”.

The 17-items Post-Migration Living Difficulties Checklist (47, 48) adapted to the Swiss context (49) was used to assess PMLD. Items include for example: “communication difficulties”, “separation from the family”, “difficulties obtaining financial assistance”. Each item measures the presence and importance (0 = not a problem, to 4 = very serious problem) of post-migration stressors in the past 12 months. In the present study, items with a score of 2 or above (at least moderately serious problem) were summed up to a total count of PMLD.

Emotion regulation was assessed using the 18-items short form of the Difficulties in Emotion Regulation Scale (DERS) (39), which has proved to maintain the psychometric properties of the original 36-items measure (50, 51). This self-report measure encompasses several dimensions of emotion regulation: awareness, understanding, and acceptance of emotions, as well as impulse-control and ability to engage in goal-directed behaviors regardless of the emotional state. Items include for example: “I am attentive to my feelings”, “I have difficulties making sense out of my feelings”, “When I am upset, I have difficulty getting my work done”, or “When I am upset, I have difficulty controlling my behaviors”. Each item is rated on a 5-point scale (1 = almost never, 2 = sometimes, 3 = about half the time, 4 = most of the time, and 5 = almost always). In the original version of the scale, three out of 18 items require reverse coding. However, previous experience with the scale indicated that reverse coding might have caused language difficulties (41). Thus, for items that were originally reverse coded, the wording was modified to avoid misunderstanding. We computed a mean of reported ratings ranging from 1 (“almost never”, indicating less difficulties in emotion regulation) to 5 (“almost always”, indicating more difficulties in emotion regulation). Internal consistency for this scale was *α* = 0.88 in our sample.

Perceived self-efficacy was assessed using the General Self-Efficacy (GSE) scale, a 10-items psychometric instrument measuring optimistic self-beliefs to cope with a variety of difficult demands in life (52). Items include for example: “I can remain calm when facing difficulties because I can rely on my coping abilities” or “I can solve most problems if I invest the necessary effort”. Responses are given on a 4-point scale (1 = not at all true, 2 = hardly true, 3 = moderately true, 4 = exactly true) and summed up to a final score (range 10 to 40). The GSE has been widely used and validated internationally, including in a multicultural context (53). Internal consistency for this scale was *α* = 0.94 in our sample.

### Procedure

Data were collected between 2015 and 2016 through a computer-based assessment tool, the Multi-adaptive Psychological Screening Software (MAPPS) (54). In MAPPS, participants accessed questionnaire items in their mother tongue either in a written or auditory form, in case of illiteracy. The duration of the assessment was approximately 60 min. If needed, participants received assistance from a psychiatrist, a clinical psychologist or a masters-level student of clinical psychology. Participants received 40 CHF (approx. 40 USD) for participation.

### Data Analysis

Statistical analysis was conducted using SPSS version 25. A regression analysis was performed with PGD severity score as dependent variable. Independent variables were defined *a priori* and entered into the model in two steps: first gender, age and length of stay in Switzerland as control variables, and then PTE and PMLD counts, and GSE and DERS scores as further independent variables. Pre-conditions for regression analyses were checked in terms of normal distribution of residuals, autocorrelation of residuals (Durbin-Watson test) and homoscedasticity and were found to be satisfying for PGD severity scores. Furthermore, none of the predictors showed signs of multicollinearity.

## Results

### Sample Characteristics

The characteristics of the sample are summarized in **Table 1**. The participants were mainly married men from different countries of origin including Turkey for the majority, but also Iran, Sri Lanka, Iraq, Bosnia, Afghanistan. and others. They were aged 46 on average and had been in Switzerland for a mean time of 13 years. Their level of education was rather high, yet the majority was either unemployed or pensioned. The majority had a secure visa status.

**Table 1:** Sample characteristics (N = 88).

Sociodemographic Variables	*n*	%
Age, mean (SD)	46.8	(9.6)
Gender (male)	71	80.7
Length of stay (years), mean (SD)	12.9	(7.2)
Country of origin Turkey Sri Lanka Iran Iraq Afghanistan Bosnia Others Marital Status	52 8 6 5 3 3 11	59.1 9.1 6.8 5.7 3.4 3.4 12.5
Single	16	18.2
In a relationship	7	8
Married	47	53.4
Divorced	14	15.9
Widowed	4	4.5
Education		
Primary school partially attended or completed	28	31.8
High school partially attended or completed	32	36.4
Technical college	13	14.8
Bachelor's degree	8	9.1
Post graduate degree	7	8
Employment		
Full time	11	12.5
Part time	19	21.6
Unemployed	36	40.9
Pensioned/homemaker	22	25
Visa Status		
Asylum seeker	5	5.7
Temporary visa	8	9.1
Permanent visa	23	26.1
Residency	37	42
Citizenship	15	17
**Clinical Variables**		
Probable PGD diagnosis	21	23.9
PGD severity score (range 11–55), mean (SD)	34.8	(9.0)
PTE count, mean (SD)	12.8	(4.1)
PMLD count, mean (SD)	8.87	(4.1)
DERS score (range 1–5), mean (SD)	3.17	(0.6)
GSE score (range 10–40), mean (SD)	23.5	(6.6)

PGD, prolonged grief disorder; PTE, potentially traumatic events; PMLD, post-migration living difficulties; DERS, difficulties in emotion regulation scale; GSE, general self-efficacy.

Reported trauma exposure was high, with participants reporting having experienced an average of 13 PTE. The most commonly reported PTE included “torture” (*n* = 80, 91%), “nonsexual assault by a stranger” (*n* = 69, 78%) and “combat situation” (*n* = 68, 77%). Importantly, sudden or violent interpersonal loss was very common. In fact, while “murder of a family member or friend” was reported by 83% (*n* = 73) and “unnatural death of a family member or friend” by 69% (*n* = 61) of the participants, 97% (*n* = 85) reported at least one of the two.

The number of reported PMLD per participant was high, with a mean of nine living difficulties considered at least as a moderately serious problem. The most commonly reported PMLD included “worry about family back home” (*n* = 75, 85%), “loneliness, boredom or isolation” (*n* = 69, 78%) and “being unable to return to your country in case of emergency” (*n* = 68, 77%).

Almost a quarter of the participants met the criteria for probable PGD (*n* = 21, 24%). The overall severity of PGD was intermediate, with a mean PG-13 score of 34.8 (*SD* = 9.0) (range 11 to 55).

Participants showed intermediate levels of emotion regulation, reporting difficulties on average about half the time, with a mean DERS score of 3.2 (*SD* = 0.6) (range 1 to 5). They showed rather low to intermediate perceived self-efficacy, with a GSE mean score of 23.5 (*SD* = 6.7) (range 10 to 40).

### Regression Analysis

The results of the regression analysis for severity of PGD adjusted for age, gender, length of stay, number of PTE, number of PMLD, emotion regulation, and self-efficacy are shown in **Table 2**. The final model proved to be significant (p < 0.001) and accounted for 27% of the variance. Greater PGD severity was significantly associated with both a higher DERS score and a lower GSE score. In other words, greater difficulties in emotion regulation and lower perceived self-efficacy were predictive of greater PGD severity in the regression analysis. The other factors entered in this model did not emerge as significant predictors of PGD severity.

**Table 2:** Summary of multiple regression analysis (*N* = 88).

Outcome variable	Step	Independent variable	*B*	*SE*	*Beta*	*t*	*p*	*F*	*p*	*R2*	adj. *R2*
PGD Severity	1							1.18	0.323	0.04	0.006
		Age	0.04	0.12	0.41	0.32	0.748				
		Gender	−4.06	2.46	−0.18	−1.65	0.102				
		Length of stay	0.05	0.16	0.04	0.32	0.751				
	2							5.65	<0.001	0.33	0.272
		Age	0.20	0.11	0.21	1.86	0.067				
		Gender	−2.68	2.23	−0.12	−1.20	0.232				
		Length of stay	−0.11	0.15	−0.08	−0.72	0.477				
		PTE count	−0.07	0.22	−0.03	−0.33	0.741				
		PMLD count	0.24	0.22	0.11	1.10	0.273				
		DERS score	5.28	1.49	0.35	3.53	0.001				
		GSE score	−0.45	0.13	−0.33	−3.47	0.001				

PGD, prolonged grief disorder; PTE, potentially traumatic events; PMLD, post-migration living difficulties; DERS, difficulties in emotion regulation scale; GSE, general self-efficacy.

## Discussion

### Primary Findings

This study examined the relationship between PGD severity and potential associated factors (emotion regulation, perceived self-efficacy, PMLD, PTE) in a clinical sample of refugees. The primary finding of our study was that difficulties in emotion regulation and lower perceived self-efficacy were associated with greater severity of PGD. In contrast, PTE and PMLD counts did not emerge as significant associated factors, supporting the idea that PGD severity is independent of overall trauma exposure and difficulties arising after arrival in a host country.

### PGD and Emotion Regulation

As PGD is by definition accompanied by intense emotional pain (*e.g.* sadness, guilt, anger, denial, and blame), it is not surprising that the ability to regulate emotions relates to symptom severity of PGD. In fact, previous models proposed to explain PGD symptoms have integrated emotion regulation as a main feature, along with attachment style, beliefs and appraisals, autobiographical memory, and self-identity (55–58). For instance, in Boelen and colleagues' cognitive behavioral model (56), maladaptive emotion regulation strategies defined by anxious and depressive avoidance are considered maintaining core factors of PGD symptoms. In other words, for individuals suffering from PGD, the internal emotional experience related to the loss (thoughts, feelings, memories) is perceived as so intolerable that they will rather avoid the reminders of the death and adopt behavioral patterns of inactivity or withdrawal. Similarly, in Maccallum and Bryant's cognitive attachment model (58), PGD symptoms are maintained by inflexible use of emotion regulation strategies avoiding the reality of the loss, the changed identity, and the new life situation.

Following these models, the participants in our study who showed more difficulties in understanding and accepting their emotions, as well as more difficulties modulating their emotional response and behaviors, also showed higher severity of PGD. Yet, the positive pendant of these findings is worth raising: participants with better understanding and acceptance of emotions and better modulation of emotion responses showed lower severity of PGD. Following this idea, there is reason to believe that an intervention based on awareness, observation, and nonjudgmental acceptance of emotional arousal may benefit both emotion regulation and executive control, and alleviate symptoms of grief, anxiety, and depression among bereaved individuals (59).

Although evidence about emotion regulation and PGD is limited, our findings are in line with a previous study suggesting an association between both positive and negative emotion regulation strategies, and symptoms levels of PGD, depression, and PTSD in people confronted with the death of a loved one, as well as in relatives of a long-term missing person (60). Furthermore, our findings call attention to emotion regulation as a relevant factor among refugees affected by PGD.

### PGD and Self-Efficacy

The association found between self-efficacy and PGD severity in our study is consistent with previous research suggesting the role of self-efficacy in adaptation after the loss of a loved one. In a study on bereavement coping among widows, self-efficacy was found to be an important predictor of emotional distress, as well as of psychological, spiritual, and physical well-being in the year following the loss of a husband (36). In another study examining successful long-term adaptation to conjugal bereavement, bereaved individuals who expressed self-efficacy had lower levels of grief over time than those who did not. Importantly, the relationship between grief symptoms and the individuals' positive evaluation of their abilities was independent of the evaluation of their actual actions or personal characteristics (61). In the context of bereavement following mass violence, some authors found that severity of persistent grief was predicted by posttraumatic stress symptoms through reduced self-efficacy and disrupted world views, illustrating a relationship between PGD, traumatic stress and self-efficacy (62). Considering the evidence about the role of perceived self-efficacy in recovery from a wide range of traumatic experiences (63–65), self-efficacy might be particularly important in traumatized refugees.

### PGD and Trauma Exposure

Consistent with other studies on PGD after war and displacement, the number of experienced PTE was not associated with PGD severity (21–23). However, slightly different findings were reported in a large population-based study among refugees in Australia, which found that the likelihood of PGD diagnosis was heightened as PTE exposure increased (20).

As in the nonrefugee population, the loss of a loved one by traumatic death clearly appears to be a major risk factor for developing PGD in refugees and conflicts affected populations (5, 18–20, 25, 26). In fact, the majority of the participants in our sample had experienced the murder or unnatural death of a close one. It is important to acknowledge that refugees are almost per definition exposed to repeated violence and PTE. Yet, when exploring trauma and grief in this group, it might be worth distinguishing the traumatic experience linked to the violent nature of the loss, which clearly seems to influence PGD, from the overall traumatic experiences, which may not. In other words, it is more likely to be the type of trauma that increases the risk of PGD (*e.g.* “witnessing the death of a loved one”), rather than the number of traumas experienced. Trauma checklists (such as the one used in this study) measure the *diversity* of trauma experiences. When creating a count variable, each trauma type is then equally weighted (*i.e.*, “witnessing the murder of a loved one” is weighted the same as “lack of shelter”). This probably reduces the likelihood of finding a dose–response relationship between PTE exposure and PGD.

### PGD and Post-Migration Living Difficulties

Despite the preliminary evidence for the causal role of PMLD with regard to mental health in refugees and asylum seekers (49, 66–68) and despite the high amount of reported PMLD in our sample, no association was found between the number of PMLD and PGD severity. However, evidence regarding PMLD and PGD is scarce.

According to previous studies, some specific PMLD such as adaptation difficulties, experiencing discrimination and being in receipt of government benefits, might be related to PGD diagnosis, yet there seems to be no significant relationship with the total number of PMLD (20, 26). Similarly, a study among asylum seekers in Germany showed that permanent visa status was associated with less severe PGD symptoms (23), yet the relationship between other PMLD and PGD was not explored. Finally, one study among West Papua refugees displaced in Papua New Guinea suggested an increased vulnerability for PGD due to stressful living conditions including food and water shortage, geographical isolation, and lack of services, as well as fear of hostile incursion from across the nearby border. This last example reminds us that PMLD may vary a lot from one host country to another, depending on the social, political, and economic context. For instance, the majority of refugees in our sample had a secure visa status and were *a priori* concerned by loneliness and boredom or by the situation in their country of origin and for their family rather than by fear for their immediate safety or vital needs.

### Thoughts on the Prevalence of PGD

Our study further revealed that nearly one out of four participants fulfilled the diagnostic criteria for PGD. Although the time elapsed since their loss was unknown, it is likely that they had been experiencing grief-related symptoms for several years, as the participants in our sample had been in Switzerland for a mean of 13 years. This finding is important for clinical practice as it highlights that special attention should be paid to grief-related symptoms in refugees seeking psychological treatment, even several years after arrival in their host country.

Compared to the current literature, the prevalence of PGD in our sample appeared to be rather low. Nevertheless, the limited number of studies on PGD among refugees and the heterogeneity of PGD assessment make it difficult to compare results. For example, a systematic review among adult refugees exposed to bereavement and trauma reported a prevalence of PGD of 33.2% (range 15–54) (14). Yet, only four highly heterogeneous studies were included in the meta-analysis on prevalence, indicating that these results should be interpreted with caution. This issue was also highlighted in a review of 24 studies that assessed grief disorder in refugees and post-conflict samples (15). The authors reported highly diverse rates of PGD depending on the standard measures employed, with an average of 32% (range 8–69) of participants scoring in the disordered or severe range. Furthermore, they reported that studies using culturally adapted assessment approaches revealed significant levels of culturally specific symptoms and even higher rates of disordered grief (range 31–76%).

As participants in our sample came from several countries of origin, it was not possible to adapt our PGD measure to each specific cultural background. This bias may have lowered our prevalence rate. Moreover, the PG-13 scale used in our study does not allow for clinical judgement regarding the expected social, cultural or religious norms in an individual's context. A caveat stating that the grief-related symptoms must clearly exceed sociocultural norms has recently been added to the ICD-11 criteria, which otherwise share the core features of the PGD-2009 criteria (6).

Previous studies using the PG-13 scale among refugees and conflict-affected populations have reported rates of PGD ranging from 8 to 34.6% (19, 23, 69–71). Again, a comparison is difficult as respective study samples differ substantially from ours in terms of age, gender, or setting.

### Further Findings

Interestingly, the majority of the participants in our sample were male. This is in contrast with most studies on PGD in adults and in particular those on the validation of the criteria for PGD, which include a high proportion of female (10, 72). As expected, based on previous studies in refugees and conflict survivors (19, 23), PGD severity was independent from gender in our model. Yet considering that the female might be at higher risk for PGD following violent loss (73), the high amount of male in our sample might have lowered the prevalence of probable PGD diagnosis. Furthermore, gender-specific processes or social norms might influence the way that individuals express grief symptoms (74, 75). These aspects need further exploration.

### Clinical Implications

Considering the high prevalence of PGD among refugees and its association with psychological comorbidity and functional impairment (20, 71), early detection and adequate treatment are essential. Left untreated, individuals suffering from PGD might remain with high levels not only of psychological (20, 76), but also of physical distress (69, 70, 73), as well as with an increased risk of suicide (70, 73, 77).

As psychological impairment may severely compromise the already challenging process of social integration in a host country, including language proficiency and financial independence (49), untreated PGD might represent not only an individual inconvenience but also an economic burden for host societies. A recent large population study, however, found that almost half of the refugees with probable PGD had not received psychological assistance (20). Explanations proposed by the authors were not only often-low help seeking among people with PGD and avoidance of confrontation to grief-related emotions, but also low mental health care utilization by refugees possibly due to stigma or lack of knowledge about referral opportunities. In the Swiss context, socio-cultural and structural barriers to accessing mental health care by refugees and asylum-seekers may include language difficulties, problems related to medical or authority gatekeepers, lack of resources, lack of awareness about health, fear of stigma, and mismatch between the local health system and perceived needs of refugees and asylum seekers (78). Over and above addressing these barriers to psychological assessment and treatment in general, our findings underline the importance of independent grief assessment, which is often not included in screening instruments for refugees (79, 80). Despite the confounding effect of experiencing both traumatic events and interpersonal loss, refugees and conflict-affected populations seem to present distinctive symptom profiles of PGD and/or PTSD (18, 21, 26).

Another implication of our findings is that they highlight potential directions for treatment interventions. Current effective treatment of PGD relies on Cognitive Behavioral Therapy (CBT), along with exposure techniques (4, 81–83). Such techniques rely on the hypothesis that directing patients to access their emotional memories can reduce avoidance of key-memories of the deceased and facilitate adaptation by restructuring core cognitions that may complicate grief response (4). Though interventions are available, knowledge about underlying factors predicting treatment response is limited (84). Moreover, these interventions have not been tested among refugees.

Given the finding that difficulties in emotion regulation and lower perceived self-efficacy are associated with greater PGD severity, it may be useful to apply techniques focusing on improving emotion regulation and perceived self-efficacy in the treatment of PGD. Such approaches have already been examined in the treatment of trauma-related disorders. For example, the Skills Training in Affective and Interpersonal Regulation (STAIR) Narrative Therapy, an intervention focusing on emotion regulation and interpersonal skills to promote resilience, has shown promising results in alleviating trauma-related distress and improving emotional and social impairments among trauma survivors (85). Similarly, refugee torture survivors showed greater distress tolerance on trauma reminders after receiving an intervention aiming to enhance perceived self-efficacy (38). The potential effects of such interventions on PGD in refugees should be targeted by future studies.

### Limitations

This study has several limitations. First, the relatively small sample size may have reduced the statistical power of the analysis. Second, the use of self-report measures may have led to several biases (*e.g.* interpretation of the question, introspective ability, honesty of response), whereas standardized clinical interviews could have strengthened our results. Third, as participants came from various cultural backgrounds, it was not possible to use measures validated among all cultural groups. In particular, it was not possible to use a culturally adapted approach when assessing PGD. Fourth, the cross-sectional design of the study prevents establishing any causality between perceived self-efficacy, emotion regulation and PGD. Fifth, information on loss-related characteristics was sparse, which limits the interpretation of the findings. Sixth, due to the clinical nature of our sample, interference of our findings with psychotherapeutic and pharmacological treatment cannot be ruled out. Moreover, as our findings were based on refugees in psychological treatment, they may not be extendable to the general refugee population.

## Conclusions

This study is one of the very few investigating PGD among refugees residing in western countries. Our findings highlight the relationship between both emotion regulation and perceived self-efficacy and the severity of PGD among refugees receiving psychological treatment in Switzerland. We consider the concepts of perceived self-efficacy and emotion regulation particularly relevant for clinical practice as they relate to subsequent behaviors and may constitute key targets for treatment intervention. Enhancing perceived self-efficacy and emotion regulation may be useful to improve treatment response of PGD in treatment-seeking refugees. Such intervention effects should be examined in future studies.

## Data Availability Statement

The datasets generated for this study are available on request to the corresponding author.

## Ethics Statement

The studies involving human participants were reviewed and approved by KEK‐ZH‐Nr. 2011‐0495 and KEK-BE_Nr. 152/12. The patients/participants provided their written informed consent to participate in this study.

## Author Contributions

OL was involved in the analysis and interpretation of data, the drafting and the revision of the manuscript. MS and NM were involved in the conception and design of the study, the acquisition, analysis, and interpretation of data, and the drafting and revision of the manuscript. JS was involved in the analysis and interpretation of data and contributed to the manuscript. RK was involved in the drafting and revision of the manuscript. AN, RB, and US designed the study and contributed to the manuscript. All authors read and approved the final manuscript.

## Funding

The study was supported by the Parrotia Foundation, the Swiss Foundation for the Promotion of Psychiatry and Psychotherapy, the Swiss Federal Office for Migration (3a-12-0495), and the Swiss Federal Office for Health (12.005187). Angela Nickerson was supported by a National Health and Medical Research Council Clinical Early Career Fellowship (1037091). The funding source played no role in the choice of the study design, in the collection, analysis, and interpretation of data, in the writing of the report, or in the decision of submitting the paper for publication.

## Conflict of Interest

The authors declare that the research was conducted in the absence of any commercial or financial relationships that could be construed as a potential conflict of interest.

The handling editor declared a past collaboration with one of the authors, AN.
